# Selective Fe(ii)-fluorescence sensor with validated two-consecutive working range using N,S,I-GQDs associated with garlic extract as an auxiliary green chelating agent

**DOI:** 10.1039/d2ra01381a

**Published:** 2022-05-12

**Authors:** Nipaporn Pimsin, Chayanee Keawprom, Yonrapach Areerob, Nunticha Limchoowong, Phitchan Sricharoen, Prawit Nuengmatcha, Won-Chun Oh, Saksit Chanthai

**Affiliations:** Materials Chemistry Research Center, Department of Chemistry and Center of Excellence for Innovation in Chemistry, Faculty of Science, Khon Kaen University Khon Kaen 40002 Thailand sakcha2@kku.ac.th; Department of Industrial Engineering, School of Engineering, King Mongkut's Institute of Technology Ladkrabang Bangkok 10520 Thailand; Department of Chemistry, Faculty of Science, Srinakharinwirot University Bangkok 10110 Thailand nuntichoo@gmail.com; Department of Premedical Science, Faculty of Medicine, Bangkokthonburi University, Thawi Watthana Bangkok 10170 Thailand phitchan.s@gmail.com; Thailand Institute of Nuclear Technology (Public Organization) Ongkharak Nakhon Nayok 26120 Thailand; Creative Innovation in Science and Technology and Nanomaterials Chemistry Research Unit, Department of Chemistry, Nakhon Si Thammarat Rajabhat University Nakhon Si Thammarat 80280 Thailand; Department of Advanced Materials Science and Engineering, Hanseo University Seosan Chungnam Republic of Korea

## Abstract

The goal of this work was to use the pyrolysis process to synthesize graphene quantum dots doped with garlic extract (as N,S-GQDs) and simultaneously co-doped with iodine (as I-GQDs). XPS, HR-TEM, FE-SEM/EDX, FT-IR, fluorescence, and UV-visible absorption spectroscopy were used to characterize the N,S,I-GQDs and analyze their morphological images. The quantum yield of N,S,I-GQDs was found to be 45%, greater than that of undoped GQDs (31%). When stimulated at 363 nm, the N,S,I-GQDs display a strong fluorescence intensity at a maximum wavelength of 454 nm. Using N,S,I-GQDs as a fluorescence quenching sensor for screening tests with various metal ions, it was discovered that they are extremely selective towards Fe^2+^ over Fe^3+^ and other ions. Thus, solution pH, concentration of N,S,I-GQDs, quantity of garlic extract, EDTA and AgNO_3_ concentration as masking agents, reaction duration under ultrasonic aid, and tolerable limit of Fe^3+^ presence in the target analyte were all optimized for Fe^2+^ detection. A highly sensitive detection of Fe^2+^ was obtained using a linear curve with *y* = 141.34*x* + 5.5855, *R*^2^ = 0.9961, LOD = 0.11 mg L^−1^, and LOQ = 0.35 mg L^−1^. The method precision, given as RSDs, was determined to be satisfactory at 1.04% for intra-day analysis and 3.22% for inter-day analysis, respectively. As a result, the selective determination of trace amounts of Fe^2+^ in real water samples using such labile multi-element doped GQDs in conjunction with garlic extract as a green chelating agent to maintain its enhanced sensitivity was successfully applied with good recoveries ranging from 89.16 to 121.45%.

## Abstract 2

The goal of this work was to use the pyrolysis process to synthesize graphene quantum dots doped with garlic extract (as N,S-GQDs) and simultaneously co-doped with iodine (as I-GQDs).

## Graphical abstract

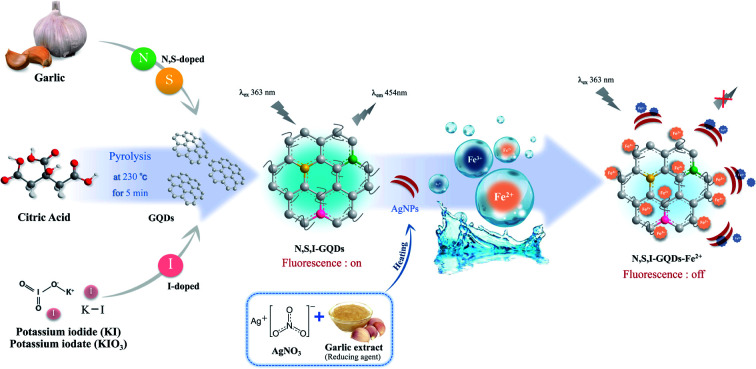

## 1. Introduction

Heavy metals are commonly discovered in high quantities in groundwater in industrially impacted regions such as the textile, metallurgical, agrochemical, and iron sectors. Their toxicity here stems from the fact that heavy metals pollute not only drinking water, but also the soil, where they accumulate in plants and animals, posing serious health risks to people. The most frequent and abundant transition metal ions in the human body are iron ions.^1^ Iron (Fe) is an important metal in biology, medicine, the environment, and industry. It is one of the most important trace elements in living bio systems, where it performs critical and flexible functions in a variety of physiological and pathological processes such as enzyme catalysis, oxygen transport, cellular metabolism, electron transfer, and DNA and RNA synthesis.^2–6^ Iron speciation is frequently linked to its role and has been discovered at a trace level. Depending on the physiological condition, it usually appears as ferrous (Fe^2+^) or ferric (Fe^3+^) ions.^7^ Iron ions in sufficient amounts are essential for optimal health. Excess iron ions in the human body can lead to major issues such as kidney and liver damage,^8,9^ neurological illnesses,^10,11^ malignancies, hemochromatosis, and crucial organ malfunction. As a result, determining iron characteristics is critical for the early detection and diagnosis of many disorders. Furthermore, measuring iron concentrations in water samples is critical for environmental safety.^12^ Solid phase extraction,^13^ high performance liquid chromatography (HPLC),^14^ inductively coupled plasma mass spectrometry (ICP-MS),^15^ fiber-optic chemosensor,^16^ inductively coupled plasma optical emission spectrometry (ICP-OES),^17^ and slotted quartz tube-flame atomic absorption spectrometry (SQT-FAAS)^18,19^ are currently used to determine Fe^2+^ and Fe^3+^ ions. Although these techniques are very sensitive and selective, they need time-consuming sample preparation and pre-concentration processes, as well as costly apparatus and experienced people. As a result, developing simple analytical procedures for the identification of Fe^2+^ and Fe^3+^ ions in natural materials remains difficult. Several fluorescence sensors have been developed in recent years for qualitative and quantitative studies due to their experimental speed, simplicity, and ability to give a selective and sensitive approach for iron speciation.

Light-emitting quantum-sized graphene quantum dots (GQDs) have lately received a lot of interest as a replacement for quantum dots (QDs) in a number of applications.^20–25^ Because of their ease of manufacture, high quantum yield, sensitivity, selectivity, biocompatibility, adjustable emission, cheap cost, strong photo stability, and nontoxicity, GQDs are intriguing in the field of chemical sensing for the detection of metal ions.^26^ In addition to the qualities listed above, GQDs offer exceptional optical, electrical, and thermal properties.^27–30^ It is one of the most common solutions for detecting harmful metal ions when combined with sensors. As a result, GQDs must enhance the surface for high selectivity, strong sensitivity, different detection limits, easy-to-use sensors, and good stability.

Garlic (*Allium sativum* L.) has been regarded as a great spice with powerful therapeutic powers by people all over the world since ancient times.^31^ The major ingredient in garlic extract is allicin; garlic's therapeutic benefits rely on organosulfur compounds obtained mostly from alliin, which has an inhibitory impact on the development of many bacteria and fungus. Garlic has been used to treat a range of diseases, including heart disease, infections, and cancer prevention.^32–34^ Other sulfur-containing phytoconstituents found in garlic include ajoenes, vinyldithiins, and amino acids such as arginine, glutamic acid, aspartic acid, and leucine.^35–37^ We introduce a new fluorescence sensor probe and have developed a selective method for detecting Fe^2+^ in drinking water samples in this study. Preparation of fluorescent N,S,I co-doped GQDs, comprised of garlic extract doped GQDs and I-GQDs, utilizing a simple, green, and low-cost one-pot pyrolysis technique using garlic, citric acid, potassium iodide (KI), and potassium iodate (KIO_3_) as the precursor. However, nearly no data on such I-GQDs have been published, however iodine supplementation can affect the optical characteristics of the GQDs with great selectivity and stability for the detection of Fe^2+^. Furthermore, the best conditions for each garlic extract and N,S,I-GQDs content, pH of the solution, masking agent, and interfering ions were thoroughly examined. The devised approach was then used to determine the iron(ii) ion in actual water samples.

## 2. Experimental

### 2.1. Materials and reagents

The chemicals utilized were all of analytical grade. Carlo Erba supplied the citric acid and sodium hydroxide (Italy). Sigma-Aldrich provided iron(ii) sulfate heptahydrate, iron(iii) chloride hexahydrate, silver nitrate, ethylenediaminetetraacetic acid (EDTA), and boric acid (H_3_BO_3_) (Germany). Acetic acid (Merck, Germany), potassium iodate (KIO_3_) (QRec, New Zealand), and phosphoric acid (H_3_PO_4_) (BDH, England) were also utilized. Ajex Finechem supplied the paraffin oil (Australia). Throughout the studies, deionized water (Simplicity Water Purification System, Model Simplicity 185, Millipore, USA) was utilized.

### 2.2. Apparatus and instruments

The principal instrument was a spectrofluorophotometer (Shimadzu RF-5301PC, Japan) with excitation and emission slit widths of 5 nm. Agilent's UV-visible spectrophotometer model 8453 was used (Germany). A pH meter UB-10 UltraBasic (Denver, USA), an analytical balance (Model LX 220A, Precisa, Thailand), a quartz cell with a path length of 1 cm (Fisher Scientific, USA), and an ultrasonic cleaner (Model VGT-2300, GT SONIC, Hong Kong) were also utilized. On a TENSOR27 system Fourier transform infrared spectrometer, an attenuated total reflectance-Fourier transform infrared (ATR-FTIR) spectroscopic observation was made (Bruker, Germany). The technique of high-resolution transmission electron microscopy (HR-TEM, Electron gun: Schottky field emission type electron gun) was employed. A HITACHI S-3000N scanning electron microscope was used to collect EDX spectra (SEM, Hitachi Co. Ltd, Japan). For the citric acid pyrolysis, a round bottom flask (Pyrex®, England) and a heated plate with a magnetic stirrer in conjunction with a paraffin oil bath were used.

### 2.3. Synthesis and characterization of GQDs and N,S,I-GQDs

Pyrolysis was used to prepare garlic extract doped/iodine co-doped graphene quantum dots (N,S,I-GQDs). Citric acid (0.9 g) was put into a 100 mL round bottom flask. The flask was heated to 230 °C using a paraffin oil bath for 5 min. The citric acid was slowly liquated with its yellow color. Then garlic extract (1 mL), KI (0.15 g) and KIO_3_ (0.15 g) were added into the flask and thermally treated for 5 min. After that, NaOH solution (0.25 M, 50 mL) was mixed with the liquid at room temperature under continuous stirring for 30 min. The achieved N,S,I-GQDs solution was stored at 4 °C until use.

### 2.4. Fluorescence measurement

The N,S,I-GQDs fluorescence was measured in Britton–Robinson buffer solution at pH 8. In a 10 mL volumetric flask, 100 mgL^−1^ of N,S,I-GQDs solution was well mixed for the following tests. Then, at room temperature, different amounts of Fe^2+^ were added to an aliquot of the N,S,I-GQDs solution in a 10 mL final volume. The Fe^2+^ fluorescence sensor of each N,S,I-GQDs solution was instantly recorded at *λ*_ex_/*λ*_em_ = 365/455 nm. Their spectral data were utilized to generate a quenching calibration curve for Fe^2+^ in the presence of AgNO_3_ solution as a masking agent for Fe^3+^ in the sample solution, if necessary.

### 2.5. Real sample analysis

The applicability of the suggested approach for actual water samples including tap water and drinking water was evaluated using an N,S,I-GQDs-based fluorescence sensor for Fe^2+^ detection concurrently in an artificial system. Tap water samples were obtained from the Khon Kaen region and placed in plastic bottles that had been prepared with 1% (v/v) dilute nitric acid. 1 mL of water and 1 mL of N,S,I-GQDs (100 mg L^−1^) solution were put into a 10.0 mL volumetric flask for this process. Prior to fluorescence measurement, each of the sample mixes was spiked with three concentration levels of Fe^2+^ standard solution (10, 25, and 50 mg L^−1^) as was done for its recovery investigation.

## 3. Results and discussion

### 3.1. Characterization of the as-synthesized GQDs and N,S,I-GQDs

#### 3.1.1 FT-IR and XPS of GQDs and N,S,I-GQDs

**Fig. 1:**
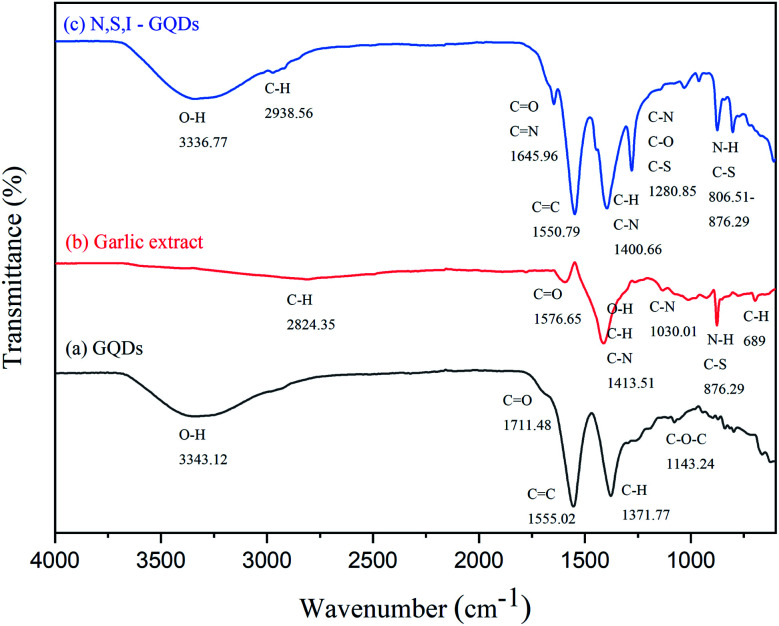
FT-IR spectra of GQDs (a), garlic extract (b) and N,S,I-GQDs (c).

FTIR and X-ray photoelectron spectroscopy were used to examine the N,S,I-GQDs produced by one-pot pyrolysis for functional groups, elemental atoms, and chemical bonds (XPS). The FTIR spectral analysis (Fig. 1a–c) confirmed that the peaks at around 3300, 2900, 1650, and 1200 cm^−1^ are assigned for the vibrations of O–H, C–H, C=O/C=N, and C–O bonds,^38,39^ and these results confirm that the garlic extract doped with I-GQDs were successfully synthesized by citric acid pyrolysis. Fig. 1c depicts the FTIR spectrum of N,S,I-GQDs, with the widened band at 800–1200 cm^−1^ corresponding to the stretching vibrations of C–S, C–N, and N–H.^40^

**Fig. 2:**
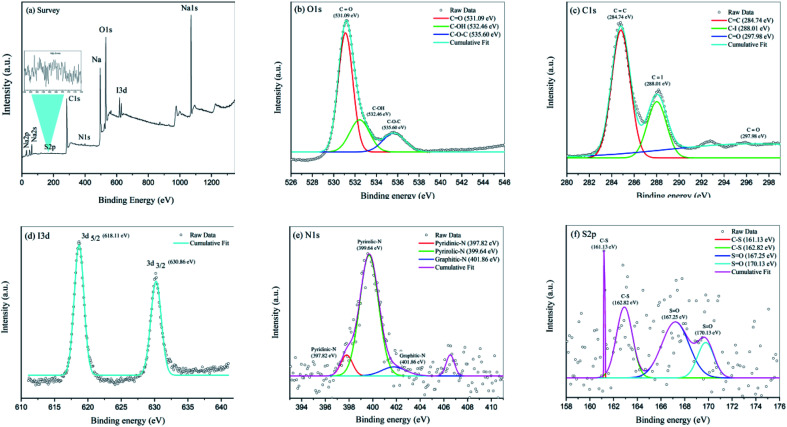
(a) XPS survey spectrum of N,S,I-GQDs, (b) high resolution of O1s XPS spectra, (c) C 1s XPS spectra, (d) I 3d XPS spectra, (e) N 1s XPS spectra and (f) S 2p XPS spectra.

The elemental compositions, carbon bonding, oxygen bonding, and iodine bonding topologies of N,S,I-GQDs were determined using X-ray photoelectron spectroscopy (XPS). The survey spectrum of the N,S,I-GQDs is shown in Fig. 2a. Peaks at 282.56, 528.49, and 619.90 eV may correspond to the binding energies of carbon (C 1s), oxygen (O 1s), and iodine (I 3d), respectively. The XPS spectra of O 1s (Fig. 2b) reveals the presence of C=O, C–OH, and C–O–C functional groups, with deconvoluted binding energies of 531.09, 532.46, and 535.60 eV, respectively.^41^ This clearly suggested that iodine was doped into GQDs effectively. The N,S,I-GQDs' high-resolution I3d spectra may be divided into two peaks at 618.65 eV (3d_5/2_) and 630.20 eV (3d_3/2_) (Fig. 2d),^42^ a substantial number of oxygen functional groups were detected in the C 1s high-resolution XPS spectra of N,S,I-GQDs (Fig. 2c), indicating inadequate carbonization during the pyrolysis of citric acid and iodine. Furthermore, two additional peaks were found in the C 1s spectra of N,S,I-GQDs at 284.74, 288.01, and 297.98 eV, corresponding to the C=C, C–I, and C=O bonds, respectively. The N,S,I-GQDs high-resolution N 1s spectra may be divided into three peaks at 397.80 eV (pyridinic-N), 399.66 eV (pyrrolic-N), and 406.56 eV (graphitic-N) (Fig. 2e). The S 2p spectra of N,S,I-GQDs (Fig. 2f) has two peaks centered at 161.1 and 169.82 eV, indicating that S exists in two forms. The former peak may be deconvoluted into two different components at 161.1 and 162.8 eV, which agree with –C–S–C– locations, while the later peak can be fit with two components at 167.3 and 169.82 eV, which agree with O=S=O. According to XPS spectra, the N,S,I-GQDs contain 58.67% carbon, 38.67% oxygen, 1.41% nitrogen, 0.85% iodine, and 0.41% sulfur.

#### 3.1.2 HR-TEM and FE-SEM/EDX image analysis

**Fig. 3:**
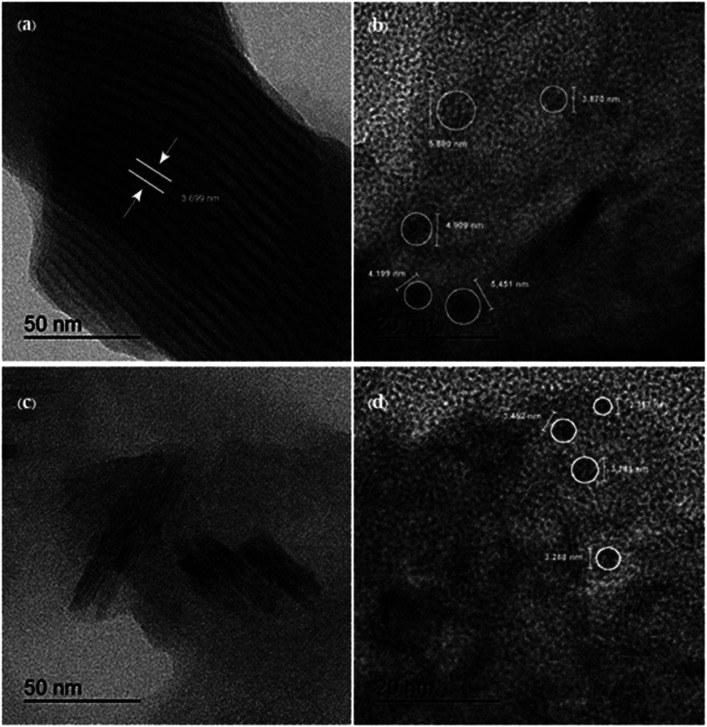
(a and b) High resolution transmission electron microscopy (HR-TEM) images of I-GQD and (c and d) HR-TEM image of N,S,I-GQDs.

**Fig. 4:**
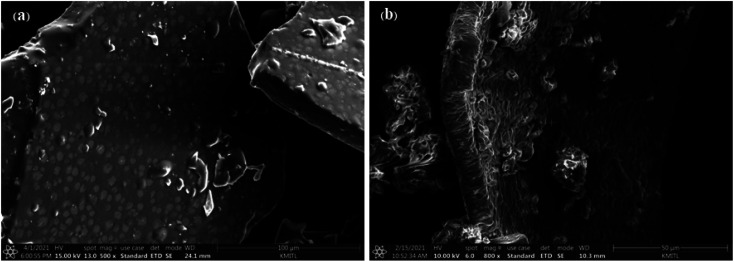
FE-SEM images of (a) GQDs (b) N,S,I-GQDs.

**Fig. 5:**
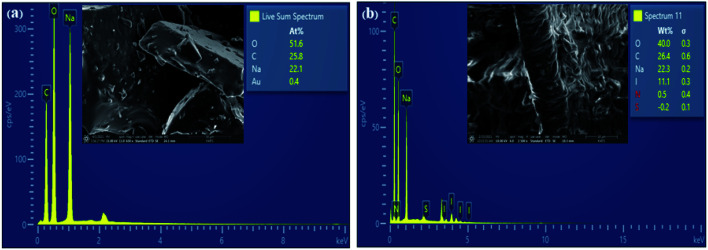
FE-SEM/EDX images of (a) GQDs (b) N,S,I-GQDs.

High resolution transmission electron microscopy (HR-TEM) was used to examine the structural and morphological information of the generated samples. In comparison to Fig. 3a and b, the I-GQDs exhibit a prominent lattice with a crystal plane spacing of 3.699 nm (Fig. 3c and d). I-GQDs doped with garlic extract have a spherical shape formed of rod-like crystals with an average spherical size of 2.357–3.781 nm for N,S,I-GQDs.^43^ A high-resolution surface morphology shown by a field emission scanning electron microscope (FE-SEM) highlights the variance in the surface texture of the produced GQDs and N,S,I-GQDs, suggesting element distribution. The porous aggregation of the N,S,I-GQDs wide ridges rough and uneven surfaces sheets owing to re-organization and fusing of garlic residue and iodine molecules is shown in Fig. 4b.^44^ Energy-dispersive X-ray spectroscopy (EDX) confirms the effective functionalization of N,S,I-GQDs (Fig. 5b).

Energy-dispersive X-ray spectroscopy confirms the elemental compositions of GQDs and N,S,I-GQDs (EDX). The GQDs had the following elements: 51.6% O, 25.8% C, and 22.1% Na (Fig. 5a). For the N,S,I-GQDs, the compositional constituents were 40% O, 26.4% C, 22.3% Na, and 11.1% I (Fig. 5b). The Na peak in GQDs and N,S,I-GQDs is caused by the NaOH solution employed in the synthesis. According to the I-GQDs, C (0.28 keV) and O (0.53 keV) occurred while a faint signal of N (0.39 keV) overlapped in between C and O, resulting in a greater compositional element of C in N,S,I-GQDs than in GQDs.

### 3.2. Fluorescence and UV-visible absorption properties of N,S,I-GQDs

**Fig. 6:**
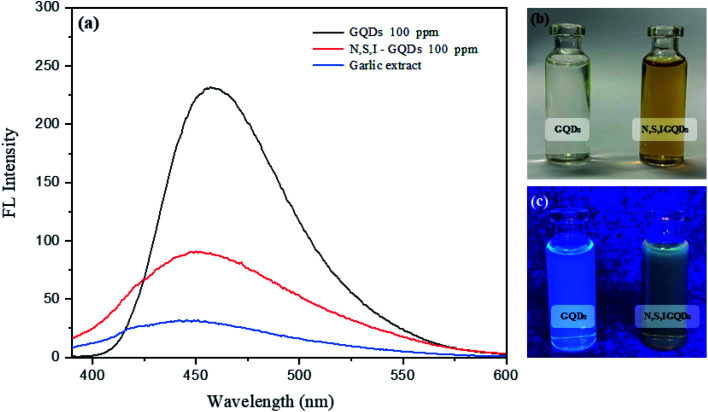
(a) Fluorescence spectra of GQDs and N,S,I-GQDs in aqueous solution slit width 3/5 at *λ*_ex_ = 365 nm. (b) GQDs and N,S,I-GQDs under visible light, and (c) blue emission of GQDs and N,S,I-GQDs under UV illumination.

**Fig. 7:**
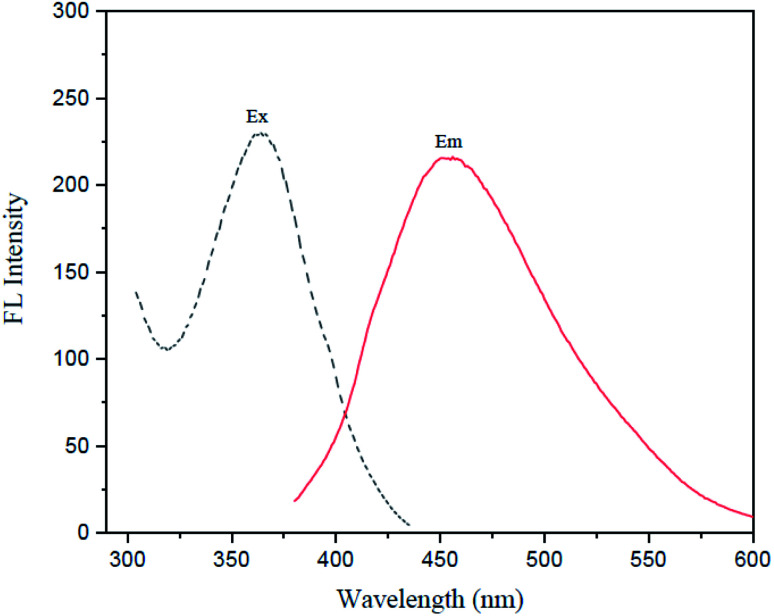
UV-vis absorption ( <svg xmlns="http://www.w3.org/2000/svg" version="1.0" width="37.000000pt" height="16.000000pt" viewBox="0 0 37.000000 16.000000" preserveAspectRatio="xMidYMid meet"><metadata> Created by potrace 1.16, written by Peter Selinger 2001-2019 </metadata><g transform="translate(1.000000,15.000000) scale(0.014583,-0.014583)" fill="currentColor" stroke="none"><path d="M80 440 l0 -40 320 0 320 0 0 40 0 40 -320 0 -320 0 0 -40z M880 440 l0 -40 320 0 320 0 0 40 0 40 -320 0 -320 0 0 -40z M1680 440 l0 -40 320 0 320 0 0 40 0 40 -320 0 -320 0 0 -40z"/></g></svg> ) and fluorescence spectra (—) of N,S,I-GQDs; *λ*_ex_/*λ*_em_ = 363/454 nm (slit width 3/5 nm), in the presence of 1.0 mgL^−1^ of Fe^2+^ containing 0.01 mL of garlic extract and 100 μM AgNO_3_.

Fig. 6 shows the fluorescence spectra of I-GQDs, garlic extract, and N,S,I-GQDs, as well as a snapshot of both GQDs and N,S,I-GQDs solution under 365 nm UV light. The GQDs and N,S,I-GQDs aqueous solutions were compared under visible light (Fig. 6b) and emitted blue light under UV irradiation (Fig. 6c). As a result, the effective doping of garlic extract-I into GQDs was confirmed. The results demonstrated that the blue emission of the GQD solution was brighter than that of the N,S,I-GQDs solution.^45^ The UV-visible absorption spectra (dotted line) and fluorescence emission spectrum of N,S,I-GQDs (Fig. 7) reveal a comparable absorption band extending from 300 nm to 600 nm as reported previously for N-doped CDs.^46^ When stimulated at 365 nm, the N,S,I-GQDs exhibit a very strong fluorescence spectrum spanning the wavelength range of 375–600 nm, with a maximum wavelength of 455 nm (Fig. 7, red line). The N,S,I-GQDs fluorescence excitation spectrum shows a wide peak with a maximum wavelength of 365 nm (Fig. 7, dotted line).

GQDs and garlic extract-(I-GQDs) quantum yields were estimated by comparing their integrated fluorescence intensities and absorbance values to those of quinine sulfate. The quantum yield of quinine standard solution in 0.1 M H_2_SO_4_ is 0.54. Table 1 summarizes the quantum yields of several doping materials. The fluorescence quantum yields of the GQDs and garlic extract-(I-GQDs) produced were found to be 31% and 45%, respectively. The quantum yield of garlic extract-(I-GQDs) is larger than that of undoped GQDs, showing that doping of both garlic extract and I on the GQDs surface resulted in a considerable increase in the fluorescence quantum yield of the GQDs. The quantum yield (*Q*) of N,S,I-GQDs was estimated using the equation: *Q* = *Q*_R_ × [*m*/*m*_R_] × [*n*^2^/*n*_R_^2^].^47^

**Table 1:** Quantum yields of undoped GQDs, N,S,I-GQDs and quinine sulfate

Material	Quantum yield (%)
Undoped GQDs	31
N,S,I-GQDs from 2% (v/v) garlic extract doped and 0.6% (w/v) KI/KIO_3_ doped	45
Quinine sulfate	54

### 3.3. Effect of N,S,I-GQDs on the Fe^2+^–garlic extract complex

#### 3.3.1 Selectivity of metal ions on garlic extract-GQDs, and I-GQDs and N,S,I-GQDs

**Fig. 8:**
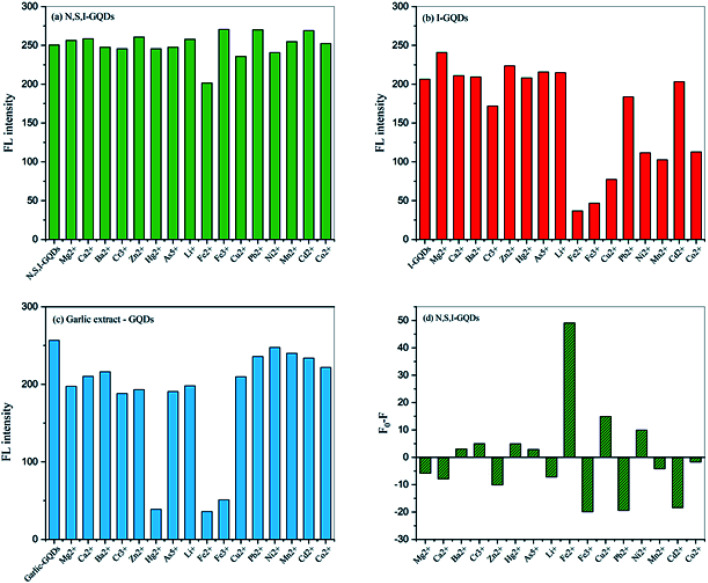
(a) Comparison of the fluorescent intensities of N,S,I-GQDs (b) I-GQDs and (c) garlic extract-GQDs using concentration of 100 mg L^−1^ in the presence of different metal ions (1 mg L^−1^) containing 0.01 mL of garlic extract and 100 μM AgNO_3_ at an excitation wavelength of 365 nm (slit width 3/5 nm) (d) their quenching efficiency of Fe^2+^ was determined by *F*_0_–*F*.

The screening test revealed that the Fe^2+^ ion had a substantial affinity for N,S,I-GQDs among the sixteen other metal ions tested, implying that it has a high potential selectivity with N,S,I-GQDs. While Fig. 8b and c show the results of metal ion detection tests employing I-GQDs and garlic extract-GQDs, respectively, they show poor potential selectivity when compared to N,S,I-GQDs. As a result, the quenching effectiveness of Fe^2+^ was clearly demonstrated as measured by *F*_0_–*F*, where *F*_0_ and *F* are the fluorescence intensities of the metal ions at various concentrations.^48^ (Fig. 8d). Because significant fluorescence quenching with Fe^2+^ was detected, the quenching test was carried out at low concentrations of Fe^2+^ ranging from 0.1 to 5 mg L^−1^.

#### 3.3.2 Effect of N,S,I-GQDs concentration

**Fig. 9:**
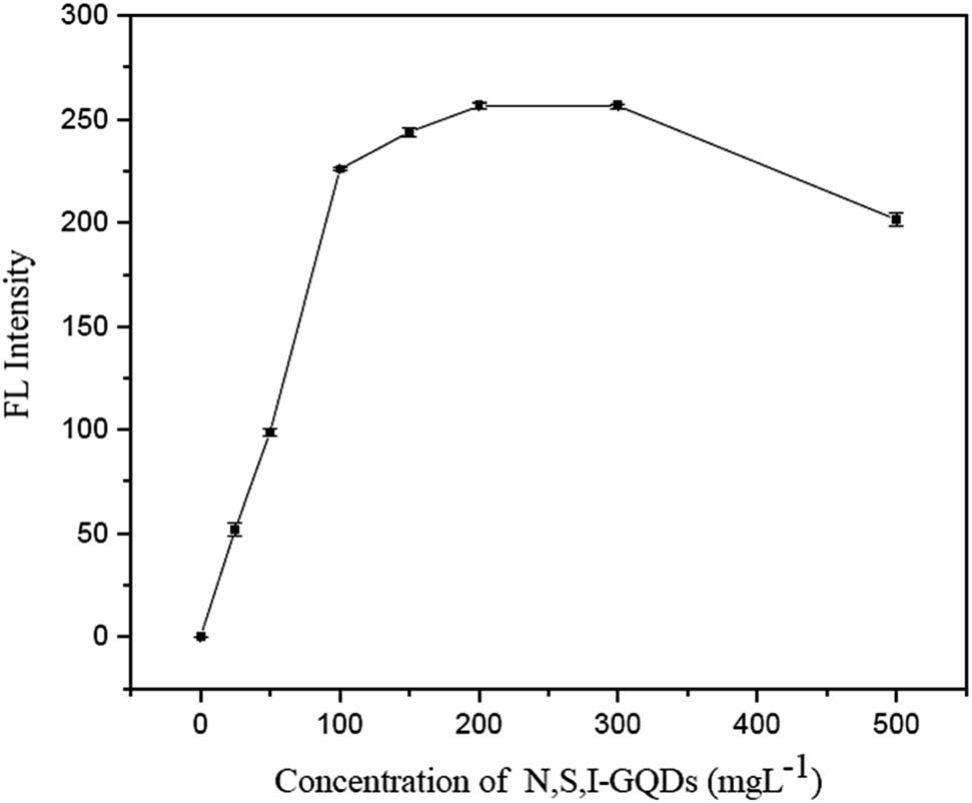
Effect concentration of N,S,I-GQDs in the presence of 0.5 mg L^−1^ of Fe^2+^ containing 0.01 mL of spiked garlic extract and 100 μM AgNO_3_ (at *λ*_ex/_*λ*_em_ = 365/455 nm slit width 5/5 nm).

The impact of N,S,I-GQDs concentration was evaluated using fluorescence intensity. According to the results, the quantity of N,S,I-GQDs required for the full reaction of Fe^2+^ ranged from 100 to 500 mg L^−1^ (Fig. 9), with no discernible difference in the fluorescence intensity of the garlic extract-(I-GQDs). As a result, it was decided to utilize 100 mg L^−1^.

#### 3.3.3 Effect of EDTA as general masking agent for heavy metals

**Fig. 10:**
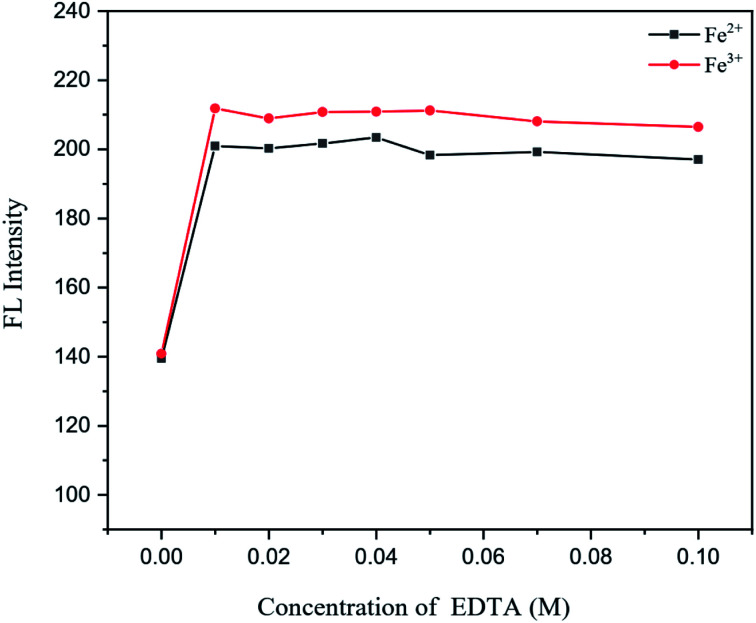
Effect of EDTA in the presence of 0.5 mg L^−1^ of Fe^2+^ and Fe^3+^ containing 0.01 mL of spiked garlic extract, 100 mg L^−1^ of N,S,I-GQDs and 100 μM AgNO_3_ (at *λ*_ex/_*λ*_em_ = 365/455 nm slit width 5/5 nm).

For effect study of EDTA as general masking agent of heavy metal ions presence in an aqueous sample, various concentrations of EDTA at 0.01, 0.02, 0.03, 0.04, 0.05, 0.07 and 0.10 M were added into 100 mg L^−1^ N,S,I-GQDs, 0.5 mg L^−1^ of Fe^2+^ and Fe^3+^ solutions after that adjusted to 10 mL in a volumetric flask prior to fluorescence measurement and those fluorescence spectra were recorded and plotted as shown in Fig. 10. According to the results that EDTA had no effect to Fe^2+^ and Fe^3+^ on the fluorescence intensity of N,S,I-GQDs therefore, it was chosen to use at 0.01 M.

### 3.4. Effect of AgNO_3_ as selective masking agent for Fe^3+^

**Fig. 11:**
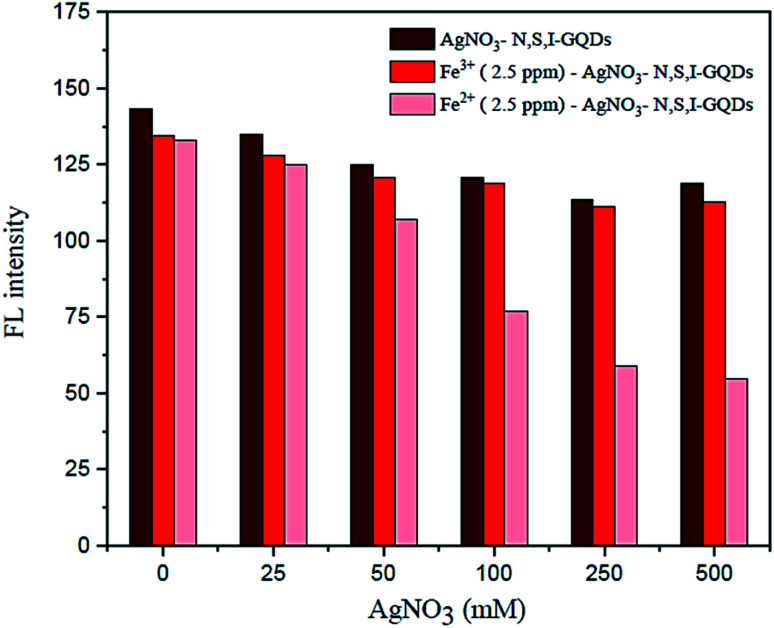
Effect of AgNO_3_ concentration of fluorescence intensity in the presence of 2.5 mg L^−1^ of Fe^2+^ containing 0.01 mL of spiked garlic extract, 100 mg L^−1^ of N,S,I-GQDs (at *λ*_ex/_*λ*_em_ = 365/455 nm slit width 5/5 nm).

Under ideal conditions, the AgNO_3_ solution in the presence of garlic extract might be reduced to get a trace of silver nanoparticles, AgNPs. By adding AgNO_3_ to the sample solution, preferably to mask Fe^3+^, the fluorescence spectrum observations were utilized to plot the quenching calibration curve for Fe^2+^. According to the results, varying the quantity of AgNO_3_ from 100 to 500 M (Fig. 11) had no discernible effect on the fluorescence intensity of the reaction conditions. As a result, 100 M AgNO_3_ was utilized in subsequent studies.

### 3.5. Effect of the garlic extract amount

**Fig. 12:**
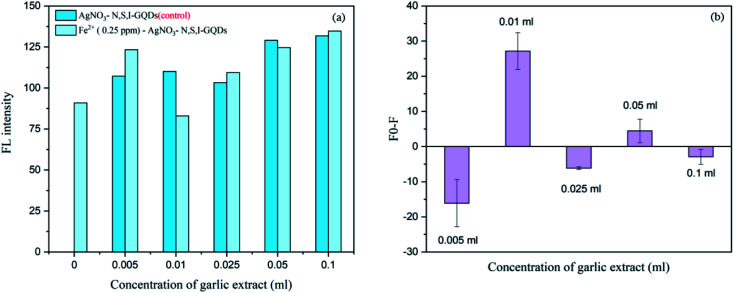
Effect of garlic extract amount on fluorescent intensity in the presence of 0.25 mg L^−1^ of Fe^2+^ containing 100 mgL^−1^ N,S,I-GQDs and 100 μM AgNO_3_ (at *λ*_ex/_*λ*_em_ = 365/455 nm slit width 5/5 nm).

Various concentrations of the garlic extract approximately spiked at 0.005, 0.01, 0.025, 0.05 and 0.1 mL were added into 100 mg L^−1^ of the N,S,I-GQDs 0.5 mgL^−1^ Fe^2+^ and 100 μM AgNO_3_. From the results (Fig. 12a and b), the fluorescence intensity and fluorescence spectra were recorded and plotted as *F*_0_–*F* were made comparison to suitable amount to induce the Fe^2+^–garlic extract (N,S–) complex formation on the surface of N,S,I-GQDs. The concentration of garlic extract was decided to be 0.01 mL based on the disclosed findings since Fig. 12b indicates the results of the experiment Fo-F at 0.01 mL greatest fluorescence intensity.

### 3.6. Effect of solution pH

**Fig. 13:**
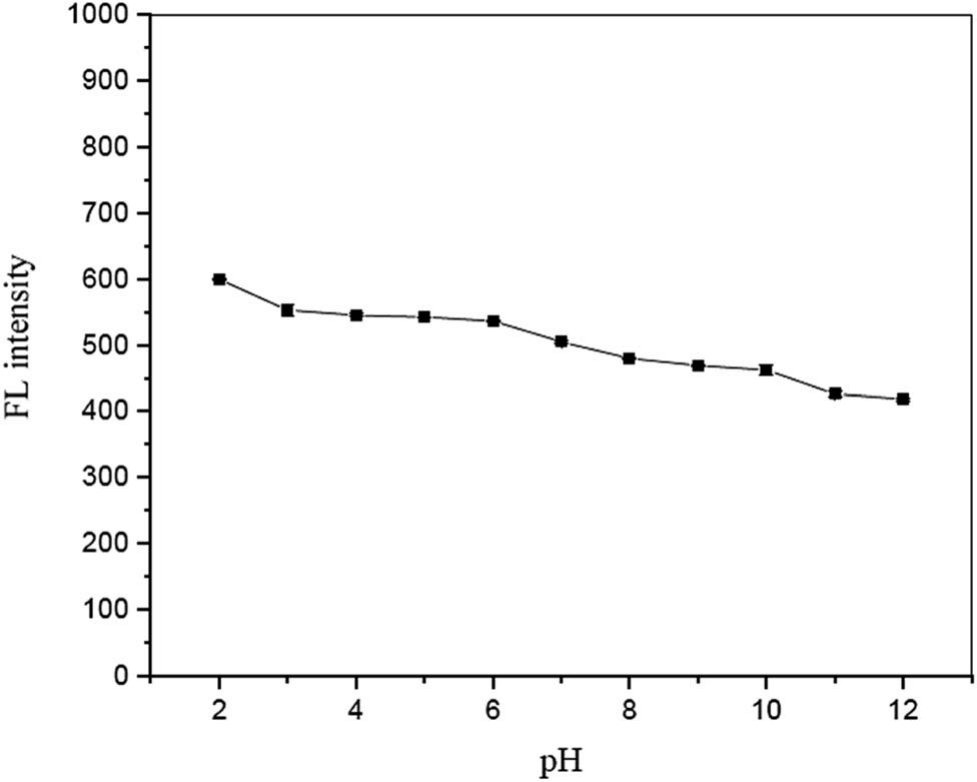
Effect of solution pH on fluorescence intensity in the presence of 2.5 mg L^−1^ of Fe^2+^ containing 0.01 mL of spiked garlic extract, 100 mg L^−1^ of N,S,I-GQDs and 100 μM of AgNO_3_ (at *λ*_ex/_*λ*_em_ = 365/455 nm slit width 5/5 nm).

The influence of solution pH on the fluorescence quenching of garlic extract-(I-GQDs) by Fe^2+^ was also investigated. The results show that a pH range of 8 to 10 (Fig. 13) had no effect on the fluorescence intensity of the N,S,I-GQDs. As a result, the pH 8 solution was chosen for future investigation.

### 3.7. Effect of tolerant limit of Fe^3+^presence in the analysis of Fe^2+^

**Fig. 14:**
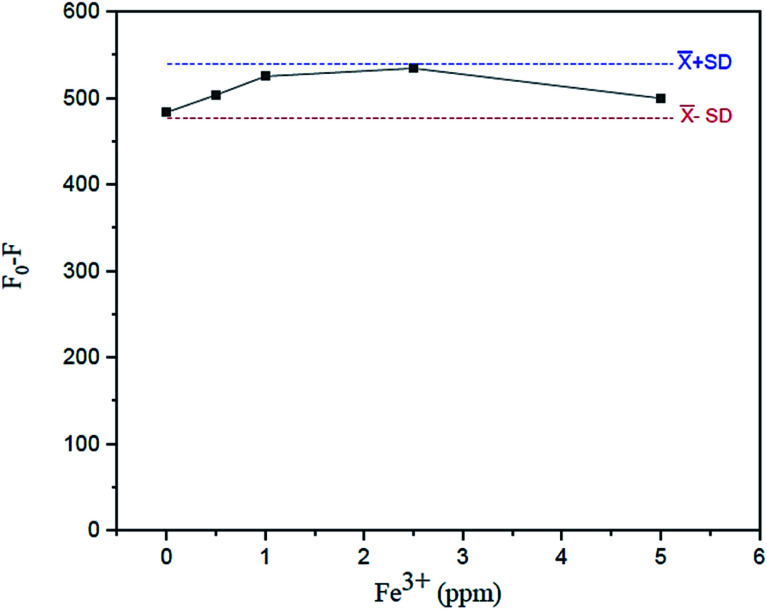
Effect of tolerant limit of Fe^3+^ on fluorescence intensity in the presence of 2.5 mg L^−1^ of Fe^2+^ containing 0.01 mL of spiked garlic extract, 100 mg L^−1^ of N,S,I-GQDs and 100 μM of AgNO_3_ (at *λ*_ex/_*λ*_em_ = 365/455 nm slit width 5/5 nm).

The effect of the Fe^3+^ tolerant limit on the measurement of Fe^2+^ in the sample solution using the suggested fluorescence quenching technique was investigated. The tolerating limit of Fe^3+^ is that to be regarded, for example, in drinking water (5 mg L^−1^ excess Fe^3+^ can be tolerated) for the measurement of Fe^2+^ was thoroughly explored by adding their known amounts (0–5 mgL^−1^ Fe^3+^), as shown in Fig. 14. It was discovered that their fluorescence signals constantly varied within an acceptable standard deviation of the mean values of *F*_0_–*F versus* Fe^3+^ concentrations.

### 3.8. Analytical features of the proposed fluorescence quenching sensor

**Fig. 15:**
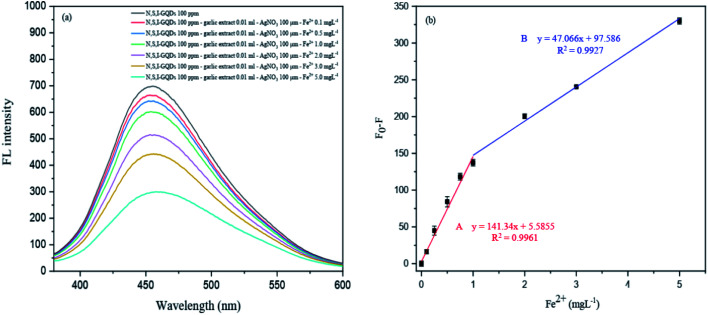
(a) The fluorescence spectra of the N,S,I-GQDs at different concentrations of Fe^2+^ and (b) the corresponding two consecutive linear plots between *F*_0_–*F vs.* concentrations of Fe^2+^ as working range A and B.

This created approach was tested for quantitative applications such as linearity, detection limit (LOD), quantification limit (LOQ), precision (% RSD), and accuracy to evaluate the fluorescence quenching sensor for Fe^2+^ detection (% recovery) (Fig. 15).

#### 3.8.1 Linearity

The experiment was carried out to evaluate the influence of Fe^2+^ concentration on the fluorescence intensity of N,S,I-GQDs for the linearity investigation. Fig. 15a depicts the fluorescence spectra of N,S,I-GQDs with varying Fe^2+^ concentrations. The spectra show that the fluorescence intensity of the N,S,I-GQDs is very sensitive to Fe^2+^ concentration. With increasing Fe^2+^ concentration, the fluorescence intensity diminishes. As a result, the quenching effectiveness of Fe^2+^ was calculated using linear (*F*_0_–*F*), where *F*_0_ and *F* are the fluorescence intensities of the blank and various metal ion concentrations, respectively. The quenching test was carried out at low concentrations of Fe^2+^ in two ranges from 0.1 to 5 mg L^−1^, referred to as Range A from 0.1 to 1 mg L^−1^ (*R*^2^ = 0.9961, regression line; *y* = 141.34*x* + 5.5855) and Range B from 1 to 5 mg L^−1^ (*R*^2^ = 0.9927, regression line; *y* = 47.066*x* + 97.586) in Fig. 15b, which shows a good fitting and these findings suggest that N,S,I-GQDs can be employed as a selective and sensitive sensor for detecting Fe^2+^ ions.

#### 3.8.2 LOD, LOQ and precision of the developed method

The limit of detection (LOD) and limit of quantification (LOQ) were calculated using the following equations;^49^ LOD = 3SD/*S* and LOQ = 10SD/*S*, where SD is the standard deviation of 3 times of blank readings and *S* is the slop of linear regression plot. Table 2 summarizes the LOD and LOQ calculations. The findings of two linear intervals for the detection of Fe^2+^; range A, Fe^2+^ detection concentration range of 0.1 to 1 mg L^−1^ (LOD = 0.11, LOQ = 0.35 mg L^−1^) and range B, Fe^2+^ detection concentration range of 1 to 5 mg L^−1^ (LOD = 0.32, LOQ = 1.07 mg L^−1^). It should be highlighted that the detection limit of Fe^2+^ observed in this study was substantially lower than Jordanian Standards and World Health Organization (WHO) recommended for drinking water quality of iron (ii) ion concentration range of 0.3–1.0 mg L^−1^^50^ and below 0.3 mg L^−1^.^51^ Their interactions with Fe^2+^ and N,S,I-GQDs are likely to lead to the aggregation of nanoparticle complexes and the tendency of the stiff structure to form stable heavy metal complexes, as well as high efficiency and sensitivity (in mg L^−1^ level). This study demonstrates that the developed method's N,S,I-GQDs substrate for Fe^2+^ detection has the lowest LOD of 0.11 mg L^−1^ (0.4 μM), when compared to selected relevant reports of Fe^2+^ trace detection by several methods, including FAAS, UV-visible/colorimetric method, and fluorescence sensor, as shown in Table 3.

**Table 2:** Limits of both detection and quantification of Fe^2+^ for the developed method

Linear curve	Linear equation	*R* ^2^	LOD (mg L^−1^)	LOQ (mg L^−1^)
A	*y* = 141.34*x* + 5.5855	0.9961	0.11	0.35
B	*y* = 47.066*x* + 97.586	0.9954	0.32	1.07

**Table 3:** Trace determination of Fe^2+^ by using fluorescence sensor compared with other research^a^

Material	Technique	LOD	Response time	Ref.
HMFluNox, HMRhoNox	Fluorescence	10 μM	60 min	52
Dicyanomethylene-4*H*-pyran and *N*-oxide (DCM-Fe)	NIR-fluorescence	4.5 μM	15 min	53
Acylated hydroxylamine moiety into the naphthalimide fluorophore	Fluorescence	0.5 μM	15 min	54
Cou-T and Rh-T	Fluorescence	0.75 μM	30 min	55
RhoNox-1	Fluorescence	0.2 μM	60 min	56
C_20_H_21_BrNP, HATU, DIPEA, DMF, RT	Fluorescence	1.03 μM	—	57
N,S,I-GQDs	Fluorescence	^a^0.4 μM	20 min	This work

a LOD 0.11 mg L^−1^ = 0.4 μM, linear range 0.1–5 mg L^−1^ = 0.36–17.98 μM.

#### 3.8.3 Precision of the developed method

The repeatability of accuracy was evaluated in terms of relative standard deviation (RSD). The repeatability (intra-day precision, *n* = 3 × 3) and reproducibility (inter-day precision, work conducted over 5 × 3 consecutive days) of the linearity slopes in two working ranges from the detection of Fe^2+^ in such acceptable settings are gathered in Table 4. Table 5 summarizes the best conditions for determining Fe^2+^ traces in drinking water and tap water samples, including the best values for chemicals, garlic extract, N,S,I-GQDs, and reaction time.

**Table 4:** Intra-day and inter-day analysis

Calibration no.	Intra-day analysis (*n* = 3)		Inter-day analysis (*n* = 5)	
	Linear		Linear	
	A	B	A	B
RSD (%)	1.04	1.05	3.23	2.72

**Table 5:** The optimum conditions of Fe^2+^ analysis in real water samples

Parameter	Analytical data
pH value (ammonium buffer solution)	8.0
Amount of garlic extract doped in GQDs (%)	2.0
N,S,I-GQDs concentration (mg L^−1^)	100
Amount of garlic extract (μL)	10
AgNO_3_ concentration (μM)	100
Reaction time under ultrasound assisted (min)	20

### 3.9. Real sample analysis

Under optimal conditions, the suggested fluorescence quenching sensor was used to determine the iron(ii) ion in various sample matrices such as drinking water and tap water. The method's correctness was confirmed by calculating the recovery study in these water samples. Each sample was spiked with three different doses of the standard Fe^2+^ solution (10, 25, and 50 mg L^−1^). The relative recoveries were then computed as follows:
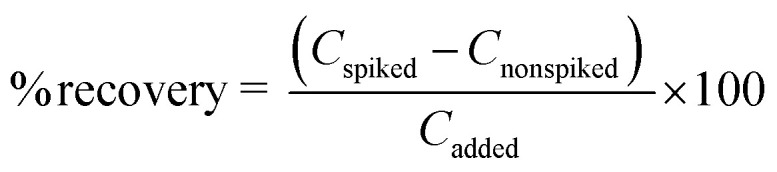
where *C*_found_, *C*_real_ and *C*_added_ denote the concentration of analyte in the real sample after addition of the known amount of standard, the concentration of an analyte in the real sample, and the concentration of the known amount of standard that was spiked in the real sample, respectively, as shown in Table 6.

**Table 6:** The Fe^2+^ contents found (*X* ± SD, *n* = 3) and its recoveries of the water samples using the N,S,I-GQDs as selective fluorescence quenching sensor

Sample	Fe^2+^ added (mg L^−1^)	Fe^2+^ found (mg L^−1^)	Recovery (%)
Drinking water 1	—	0.04 ± 11.25	—
	10	10.01 ± 3.59	99.46
	25	22.34 ± 5.83	89.16
	50	51.00 ± 2.01	101.90

Drinking water 2	—	0.22 ± 8.01	—
	10	9.97 ± 10.46	101.96
	25	27.76 ± 10.28	111.94
	50	48.97 ± 8.27	98.38

Drinking water 3	—	0.38 ± 8.01	—
	10	9.96 ± 10.46	103.43
	25	29.50 ± 10.28	119.52
	50	48.31 ± 8.27	97.40

Drinking water 4	—	0.30 ± 0.60	—
	10	9.97 ± 8.26	102.76
	25	29.84 ± 4.19	121.45
	50	48.18 ± 2.85	97.68

Drinking water 5	—	0.28 ± 4.73	—
	10	9.97 ± 7.50	102.46
	25	29.85 ± 9.44	120.50
	50	48.19 ± 6.18	96.93

Tap water 1	—	0.16 ± 3.28	—
	10	9.98 ± 5.91	101.46
	25	29.93 ± 2.42	119.93
	50	48.09 ± 7.39	96.16

Tap water 2	—	0.08 ± 9.99	—
	10	9.99 ± 1.14	100.70
	25	29.50 ± 2.81	118.32
	50	48.31 ± 0.58	96.78

## 4. Conclusion

The goal of this research was to use pyrolysis to create graphene quantum dots doped with garlic extract and concurrently co-doped with iodine. XPS, HR-TEM, FE-SEM/EDX, FT-IR, fluorescence, and UV-visible absorption spectroscopy were used to characterize and analyze the N,S,I-GQDs. When compared to undoped GQDs (31%), the quantum yield of N,S,I-GQDs was found to be 45%. When excited at 363 nm, the N,S,I-GQDs display strong fluorescence intensity at a maximum wavelength of 454 nm. Using N,S,I-GQDs as a fluorescence quenching sensor for screening tests with various metal ions, it was discovered that Fe^2+^ is extremely selective over Fe^3+^ and others. Thus, solution pH, concentration of N,S,I-GQDs, quantity of garlic extract, EDTA and AgNO_3_ concentration as masking agents, reaction duration under ultrasonic aided, and tolerable limit of Fe^3+^ presence in the target analyte were all optimized for Fe^2+^ detection. The suggested method's analytical properties were unquestionably proven, particularly with two successive working ranges of their calibration curves. The technique precision, given as RSDs, was found to be satisfactory, and therefore good accuracy was reached by utilizing spiked Fe^2+^ into drinking water and tap water samples in the recovery research. As a result, the selective measurement of trace Fe^2+^ in actual water samples employing N,S,I-GQDs linked with garlic extract as a green chelating agent to aid raise its improved sensitivity was effective.

## Conflicts of interest

The authors have declared no conflict of interest.

## Supplementary Material
